# A Multi-Parametric Device with Innovative Solid Electrodes for Long-Term Monitoring of pH, Redox-Potential and Conductivity in a Nuclear Waste Repository

**DOI:** 10.3390/s17061372

**Published:** 2017-06-13

**Authors:** Jordan Daoudi, Stephanie Betelu, Theodore Tzedakis, Johan Bertrand, Ioannis Ignatiadis

**Affiliations:** 1 Water, Environment and Eco-technologies, BRGM French Geological Survey, 45060 Orléans, France; i.ignatiadis@brgm.fr; 2 Laboratory of Chemical Engineering, Université de Toulouse III Paul Sabatier, 31062 Toulouse, France; tzedakis@chimie.ups-tlse.fr; 3 Monitoring and Data Processing Department (DRD/MTD), ANDRA French National Radioactive Waste Management Agency, 92290 Châtenay Malabry, France; johan.bertrand@andra.fr

**Keywords:** multi-parametric probe, all-solid-state electrodes, reference electrodes, pH sensor, redox potential, conductivity, galvanostatic electrochemistry impedance spectroscopy (GEIS), nuclear waste disposal monitoring

## Abstract

We present an innovative electrochemical probe for the monitoring of pH, redox potential and conductivity in near-field rocks of deep geological radioactive waste repositories. The probe is composed of a monocrystalline antimony electrode for pH sensing, four AgCl/Ag-based reference or Cl^−^ selective electrodes, one Ag_2_S/Ag-based reference or S^2−^ selective electrode, as well as four platinum electrodes, a gold electrode and a glassy-carbon electrode for redox potential measurements. Galvanostatic electrochemistry impedance spectroscopy using AgCl/Ag-based and platinum electrodes measure conductivity. The use of such a multi-parameter probe provides redundant information, based as it is on the simultaneous behaviour under identical conditions of different electrodes of the same material, as well as on that of electrodes made of different materials. This identifies the changes in physical and chemical parameters in a solution, as well as the redox reactions controlling the measured potential, both in the solution and/or at the electrode/solution interface. Understanding the electrochemical behaviour of selected materials thus is a key point of our research, as provides the basis for constructing the abacuses needed for developing robust and reliable field sensors.

## 1. Introduction

Near-neutral pH and low redox potential (E_h_) are considered to be favourable conditions for nuclear waste disposal in clay formations, because most radionuclides, including actinides, have a low solubility under such conditions [1]. Radioactive waste-management programmes today mainly focus on deep geological storage as this is currently the most appropriate strategy for ensuring the long-term safety of people and environment. “Cigeo” is the name of a future deep geological disposal facility for radioactive waste, to be built between 2020 and 2025 in France, at 500 m depth within the clayey Callovian-Oxfordian (COx) formation.

The COx formation is an assembly of mineral complexes dating back to 160 million years ago and lying at a depth of 400 to 600 m. It is a water-saturated environment with extremely low permeability, porosity and hydraulic conductivity. The temperature, pH and CO_2_ partial pressure of the COx pore-water solution are constant at 25 °C, 7.3 (±0.1) and 8 × 10^−3^ atm, respectively [2]. Anoxic conditions prevail in the COx formation. Within the mineralogical assemblage [3,4], geochemical models predict E_H_ values ranging from −180 to −200 mV, corresponding to an equilibrium between pyrite and pore-water sulphate [S^(+VI)^] concentrations, and iron-bearing phases such as Fe-bearing carbonates or nanogoethite [4,5,6,7].

The French National Radioactive Waste Management Agency (Andra) is in charge of the long-term radioactive waste management in France. Its technical specifications for the development of monitoring techniques are based on: (i) Requirements due to the specific nature of parameters that need to be measured on key thermal-hydraulic-mechanical-chemical and radiological (THMCR.) processes; and (ii) Requirements due to the minimum accuracy and long-term stability of the monitoring methods—considering that there will be little or no access for re-calibrating the sensors—for the accurate monitoring of the evolution of the near-field around the radioactive waste.

Some constraints specific to on-site conditions must be considered for developing the sensors:

1. The progressive alkalization of the COx pore water due to degradation of concrete casings (pH up to 11.7).
2. The wide range of redox potentials over the Pourbaix diagram due to: (i) gas emissions such as O_2_ due to excavation, H_2_ due to release from radioactive waste and metal corrosion, CO_2_ due to organic-matter degradation, H_2_S due to the activity of sulphate-reducing bacteria (SRB), or CH_4_ due to the activity of methanogenic bacteria; (ii) sulphide (HS^−^/S^2−^) production due to the activity of SRB; and (iii) nitrate (NO_3_^−^) production from the chemical and bacterial denitrification of the bitumen coating the concrete casings.
3. The temperature increase due to radioactive disintegration (25 °C ≤ T ≤ 90 °C).

The three key parameters for monitoring the above parameters are thus pH, conductivity and redox potential [8]. The objective was to design, create and optimize a robust multi-parameter probe for on-site monitoring of pH (±1 pH unit), redox potential (±100 mV) and electrical conductivity (±50 mS·cm^−1^), in order to ensure the long-term safety of the operation.

We present an innovative electrochemical multi-parameter probe device carrying up to 20 electrodes for such long-term monitoring. To achieve our objective, various types of electrode made of different sensitive materials were studied: a monocrystalline antimony electrode investigated as pH sensor; four silver-chloride-coated silver (AgCl/Ag) based electrodes and a silver-sulphide-coated silver (Ag_2_S/Ag) one investigated as reference electrodes and Cl^−^/S^2−^-selective electrodes; four platinum electrodes, one gold electrode and one glassy-carbon electrode, all investigated as redox-potential electrodes. AgCl/Ag and platinum electrodes were also used for conductivity measurements by galvanostatic electrochemistry impedance spectroscopy (GEIS).

We calibrated the developed sensors under conditions similar to those that will be met on-site. Overall performance, reliability and robustness were examined by electrochemical measurements at 25 °C, at atmospheric pressure and/or in a glove box (PCO_2_ = 8 × 10^−3^ atm; PO_2_ ≈ 10^−6^ atm).

## 2. Methodology

Using a multi-parameter probe offers an important advantage, because it provides redundant information, based on the simultaneous behaviour under identical conditions of different electrodes of the same material as well as that of electrodes made of different materials. This information identifies the redox reactions controlling the measured potential, both in solution and/or at the electrode/solution interface. The objective is the development of robust and “frustrated” electrodes: departing from a calibrated state, all sensors show potentiometric drift due to aging and alteration of the sensitive materials; such drift must be known and mastered for a correct interpretation of the recorded signals at any time. Knowledge of the electrochemical behaviour of the selected materials described below thus is a key point of such research, as it will help in establishing reliable abacuses.

### 2.1. Antimony-Based Electrodes for pH Sensing

Based upon reversible interfacial redox processes involving H^+^, metal/metal-oxide electrodes are a promising technology for the monitoring of pH in underground nuclear-waste disposal sites, due to their physical and chemical stability [9] concerning temperature, pressure and aggressive environments [10,11]. Metal/metal-oxide electrodes present the additional advantage of being easily miniaturized [12,13,14].

Among the metal/metal-oxide group, the antimony/antimony-oxide system, whose properties were improved by using monocrystalline antimony [15,16,17], has been the first [18,19] and most investigated (and disputed) one for pH sensing. The fact remains that it is the most commonly used system for practical pH measurements [8,20,21]. The antimony electrode potential is governed by the Sb_2_O_3_/Sb couple, since the electrode surface is spontaneously oxidized to form a thin antimony oxide film in the presence of oxygen [8,15,22].

The merits and applications of this electrode have been described for bio-medical applications [12,13,23,24,25,26], gas-sensing [27], industrial applications such as water-treatment systems, soda ash neutralization, sulphite solutions, etc. [28], or for environmental measurements [14]. It is thus of great interest to use this type of electrode for on-site pH monitoring of the near-field of a nuclear waste disposal site. However, numerous conflicting data concern the disturbance of its potential by various physical and chemical parameters [20,28,29,30]. Several authors published the influence of oxygen or certain anions, such as carbonates and phosphates, on the open circuit potential (OCP) of the antimony electrode [12], indicating that the electrode must be calibrated under conditions similar to on-site ones.

Three types of antimony-based electrodes are reported in the literature: cast electrode, plated electrode, and antimony powder electrode. The last, made of compacted of Sb^(0)^ and Sb^(+III)^ powders, has been little investigated [26], probably because of the need of a supporting electrolyte saturated by Sb_2_O_3_ to stabilize the electrode potential, which is unsuitable for continuous sensing.

Cast antimony-based electrodes in the form of a metal stick are the most common ones. The inner diameter of the glass capillary used for fabricating governs the size (macro/micro) of the electrodes. Moreover, cast antimony-based electrodes have the advantage of being relatively rugged and present a low electrical resistance, provided the electrode surface area is not too small. The electrical resistance of an electrode is an important characteristic since it is directly linked to the electrode response time [12]. Although some authors prefer to add some Sb^(+III)^ oxide to the antimony melt before casting the electrode as a cylindrical rod [20], the use of pure antimony is considered to improve the electrode characteristics with respect to reproducibility and stability of the electrode potential over long periods. The way of casting the electrode is important as well: antimony needs to be melted (T ≥ 631 °C, Sb melting point), sucked into a glass capillary and cooled down to solidify [10,12,31]. Slowly cooled electrodes are said to have a faster response than rapidly cooled ones. Furthermore, oxygen should be excluded during casting to avoid oxides in the bulk material. In addition, most methods include polishing the metal surface [12], followed by a treatment to obtain superficial oxidation, like the cumbersome oxidation method via KNO_3_ powder heated to 500 °C in a furnace for two hours under air atmosphere [10,31]. However, it must be noted that antimony spontaneously oxidizes by the oxygen contained in air [22,32]. In case of malfunction, the electrode surface can be renewed by polishing and re-oxidizing the surface. A resting period of a few days is recommended afterwards in order to relieve strain in the metal surface [33].

The literature also describes the making of antimony-coated electrodes via cathodic polarization of the sensor immersed in a solution containing Sb^(+III)^ ions [8]. Several supports such as copper, platinum and mercury-coated platinum electrodes were investigated [33], but the literature also mentions a pronounced non-reproducibility as well as fragility of the coating, limiting the lifetime of the system to about five measurements.

The first aim of our work was to investigate the reliability of monocrystalline antimony-based electrodes for their possible incorporation into a multi-parameter device for the observation and monitoring of the near field of a nuclear waste disposal site. The choice of such sensors was motivated by the high binding energy of monocrystalline antimony [23], leading to a low corrosion rate. Therefore the surface is only slowly changed and an occluding oxide is almost completely avoided [23]. The uniform binding energy promotes a general surface corrosion. Thus, only the next crystallographic plane with the same orientation and characteristics as those of the original one is exposed to corrosion. At the same time, experiments were also conducted on antimony-based screen-printed electrodes (SPEs). The reliability of SPEs has already been demonstrated for various applications in earlier papers: semi-continuous monitoring of trace metals [34], and the development of screen-printed pH electrodes based on ruthenium dioxide [32,35], cobalt oxide [36], phenanthraquinone [37] and, more recently, cerium-oxide [38]. Moreover, screen-printing is a simple and fast method for the large-scale production of reproducible low-cost sensors, which allows quick generation of reliable data [39,40]. In this study, antimony-based screen-printed electrodes were used as tools for demonstrating the reliability of monocrystalline antimony electrodes. To ensure accurate measurements, SPEs were renewed every 48 h.

### 2.2. Inert Electrodes for Redox Measurements

Platinum is considered as an inert indicator electrode for redox-potential measurements of Ox/Red systems in a solution [41,42,43]; note that platinum is easily oxidized into PtO, a well-conductive oxide that completely covers its surface. The results from platinum electrodes in well-defined concentrations of dissolved species in a solution, are stable and precise; however, in the absence of a well-defined Ox/Red system, platinum results strongly depend on its surface properties, typically the nature of the oxide covering it [41,42,43]. 

As part of subsurface redox monitoring in and around a nuclear waste disposal site, gold (Au) and glassy carbon (GC) as inert indicator electrodes were investigated in addition to platinum. Platinum differs from gold in that it has a higher exchange current density (10 mA/cm^2^ for Pt, 0.3 mA/cm^2^ for Au regarding the O_2_/H_2_O redox couple) [44]. But gold is known as a more inert material since Pt can catalyse reactions, form oxides and adsorb H_2_, which gold cannot [9]. Gold also has a greater potential range (−0.8 to 1.8 V/NHE) towards positive potentials (≈400 mV) than Pt [45]. In comparison, glassy carbon has a larger potential range (−1.0 to 1.0 V/NHE) toward negative potentials (≈−200 mV) than Au and Pt. 

### 2.3. Ag-Based Electrodes Acting as Reference or Selective Electrodes

Reference electrodes are as important as indicator electrodes [46,47,48]. Nevertheless, compared to all-solid-state indicator electrodes, the research effort into all-solid-state reference electrodes is smaller [46,48]. In their publication, Blaz et al. [48] listed the different types of existing reference electrodes. However, the interest in conducting polymer-based all-solid-state reference electrodes—equitransferent salts dispersed in polymer or compensated cationic and anionic response in polymer—grows each day [48,49,50]. 

Existing electrodes essentially use AgCl/Ag-based systems because of the invariability of their potential to pH changes, or to the presence of redox species, unless the temperature and/or the chloride ions vary. In the absence of AgCl/Ag-based systems, it is reported that the OCP of the developed electrode is influenced by redox couples, such as O_2_/H_2_O [48,50]. Whatever the investigated methodology, Ag-based electrodes predominate and seem essential. Comparatively speaking, macro-metric all-solid-state Ag-based electrodes appear stronger in the design field, as they are based on an important reserve of raw material. Moreover, Ag electrodes can either be coated by AgCl for making Ag/AgCl-based electrodes or by Ag_2_S for making Ag/Ag_2_S-based electrodes. Given the quasi-invariability of the on-site Cl**^−^** concentration (0.04 M) [5] of the COx pore-water and the very low permeability of the COx formation, the AgCl/Ag/Cl^−^ 0.04 M electrode could prove to be of primary interest for monitoring the physical- and the chemical parameter variations within and around a radioactive waste disposal site. A priori, such electrodes present a certain robustness because of: (i) the absence of feeling electrolyte; (ii) the very weak solubility product constants of AgCl (K_s_ = 10^−9.75^); and (iii) the invariability of their potential to pH changes or to the presence of redox species, unless the temperature and/or the chloride ions vary. 

In comparison with AgCl/Ag-based electrodes, an Ag_2_S/Ag-based electrode (Ag_2_S (K_s_ = 10^−49.2^) should be less soluble because of its higher pK_s_ value. In the absence of S^(−II)^, its potential will be a constant OCP. The comparison of potential values measured with both of these electrodes could help in demonstrating any changes over time of chloride and sulphide concentrations in the medium. Understanding of the electrochemical behaviour of selected materials is the second key point of our research, as it will help in constructing abacuses ([Cl^−^] = 0.04 M; S^(−II)^ = 0, in this study) that are needed for monitoring of the physical and chemical parameters of the clay barrier. 

### 2.4. GEIS for Conductivity Measurements

Geophysical methods are standard tools for obtaining information on the volumetric distribution of subsurface physical properties of rocks and fluids. One of several electrical methods that measure electrical properties of the ground is electrical resistivity (ER). In a typical ER measurement [51], four electrodes are used for measuring potential differences between pairs of electrodes, where the potentials result from a current applied between two other electrodes. By measuring at different locations, an electrical resistivity section is reconstructed as a 2D slice of the porous material [51]. Geophysical electrical methods are similar to galvanostatic electrochemistry impedance spectroscopy (GEIS) techniques. In the context of monitoring the surroundings of the radioactive waste disposal site, GEIS was selected as an alternative robust technique for conductivity measurements. A potentiostat/galvanostat was used as this investigates a larger frequency domain, in the range from mHz to MHz.

## 3. Materials and Methods 

### 3.1. Materials

#### 3.1.1. Description of the All-Solid-State Electrode Surface Materials

##### Silver Chloride/Silver-, Silver Sulphide/Silver-Based Electrodes for the Development of Reference or Specific Electrodes

A three-electrode cell (100 cm^3^) was used for creating AgCl coatings on bare Ag electrodes by oxidation. Chronopotentiometry applied a fixed anodic current density of around 0.5 mA/cm^2^ (below the chloride diffusion limited current of the oxidation of silver) between the working Ag electrodes and a platinum counter electrode, using a PAR 273A potentiostat/galvanostat (Princeton Applied Research, Oak Ridge, TN, USA). The electrodes were immersed in a 0.1 M HCl solution. The potential was monitored over the time versus a saturated calomel electrode (SCE) [52,53]. Similar experiments used silver immersed in a 0.1 M NaOH solution containing 0.1 M of Na_2_S for making Ag/Ag_2_S-based electrodes; the working electrode potential was measured with respect to a mercury/mercurous sulphate electrode (MSE).

##### Antimony-Based All-Solid-State pH Electrode

**Figure 1:**
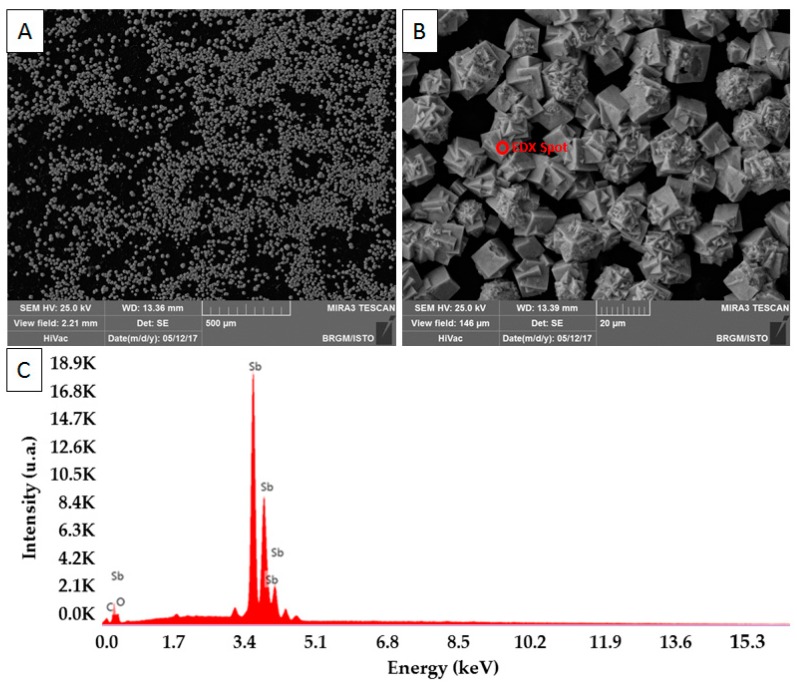
SEM-EDX analyses carried out on a freshly antimony-coated SPE. (**A**) Global view of the Sb-coated SPE working surface; (**B**) Close view of the Sb-coated SPE working surface; (**C**) EDX spectrum resulting of the analysis of the spot shown on Figure 1B.

We used a monocrystalline antimony (99.999%, m = 500 mg and d = 6.7) electrode without any pre-treatment or treatment over 16 months, in order to investigate its long-term robustness for pH monitoring. Carbon-based screen-printed electrodes (SPEs) [34,38,40,54], with a working surface of 9.6 mm^2^, were conditioned in a stirred solution containing Sb^(III)^ (10^−2^ M SbCl_3_) and HNO_3_ (pH = 0) by applying four cycles of cyclic voltammetry (potential range −0.1 V to +0.8 V, scan rate 100 mVs^−1^). The Sb crystals (Sb^3+^ + 3e^−^ → Sb) were then deposited at −0.5 V/SCE during 1600 s. These two steps were performed without removing oxygen from the solution. Figure 1 shows surface analyses carried out by scanning electron microscopy coupled to energy dispersive X-ray (SEM/EDX) on a freshly antimony-coated SPE. Experiments were performed using a TESCAN MIRA XMU scanning electron microscope (TESCAN ORSAY FRANCE, Fuveau, France). Figure 1A exhibits a representative picture of the working surface. Approximately 80% of its surface is coated with antimony. As shown on Figure 1B, shape and size (≈13 µm) of grains are homogeneous. The EDX spectrum presented on Figure 1C demonstrates the attainment of a composite material consisting in Sb-coated SPE.

Whatever the Sb-based sensors used, no preliminary anodic process took place on the electrode surface. Sb metal is slowly oxidized in air to form antimony oxide (Sb_2_O_3_) [55].

##### Platinum, Gold and Glassy Carbon as Inert Electrodes for Redox Potential Measurements

The experiments used a 10 mm disk-shaped Pt electrode (78.54 mm^2^), a 10 mm disk-shaped Au electrode (3.14 mm^2^), and a 5 mm disk-shaped glassy carbon electrode (3.14 mm^2^).

#### 3.1.2. Description of the Experimental Device and Its Components

**Figure 2:**
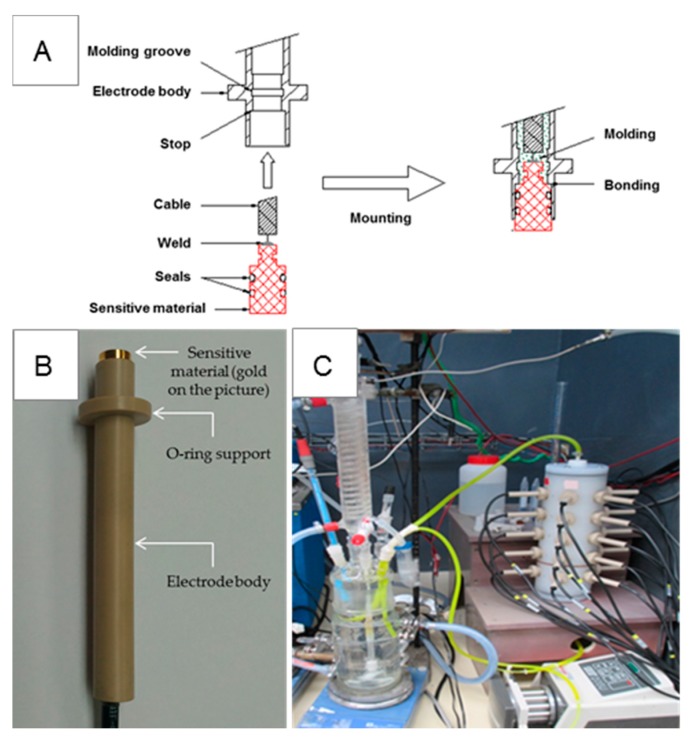
(**A**) Schematic representation of electrode construction; (**B**) Actual view of a gold-based electrode; (**C**) View of the entire experimental set-up.

A schematic representation of the electrode mounting is presented on Figure 2A. Two seals are first inserted on the sensitive element. The sensitive material is welded to the cable. Then, the assembly is slipped until the stop of the electrode body coated with glue. Finally, the body is filled with a liquid resin (molding step) which stiffens the interior and infiltrates the two molding grooves. The sensitive element is thus anchored to the body. A real view of the electrode, built according to the mounting process described above, is shown on Figure 2B. Electrode bodies are made of polyether ether ketone (PEEK), a semi-crystalline thermoplastic with excellent mechanical and chemical resistance properties, resistant to aging over several decades and stable at temperatures up to 100 °C. A maximum of 20 electrodes can be introduced within the probe-holder that is entirely made of polyvinylidene fluoride (PVDF, also called Kynar, made by Arkema, Colombes, France), which is a highly non-reactive and durable thermoplastic fluoropolymer. The internal volume of the column is about 210 cm^3^. To ensure water- and air-proofing between intra-column fluid and the exterior, each electrode has a screw-thread with stuffing box and O-ring (O-ring VITON^®^, Dieppe, France).

The whole experimental device, composed of probe holder, electrodes and the other operational elements is shown on Figure 2C. The experiments took place under dynamic conditions. The probe-holder was fed from an electrochemical cell using a peristaltic pump (Figure 2C); the fluid flowed from bottom to top of the probe-holder to avoid bubble formation and thus two-phase flow. The flow rate of the pump was set at an average of 20 mL/min to reduce long-term electrode erosion.

#### 3.1.3. Supporting Electrolytes: Buffers and Solutions

The experiments were performed at a constant temperature (25.0 ± 0.1 °C), either at atmospheric pressure or in a thermo-regulated glove box under N_2_/CO_2_ (99/1%) atmosphere (PCO_2_ = 8 × 10^−3^ atm, PO_2_ ≈ 10^−6^ atm).

pH buffers were prepared either using milliQ water (18 MΩ) or 0.1 M NaCl solution. The different conjugate acid-base pairs with their effective pH range are listed in Table 1. For all experiments investigating the influence of pH on electrode OCP, measurements were successively made by increasing then decreasing the pH values in order to highlight hysteresis effects.

Measurements were also carried out in the presence of a synthetic solution, called reconstituted COx pore water, whose major-element composition and pH at 25 °C were representative of the COx pore water (Table 2) [4,5,56,57].

### 3.2. Methods

#### 3.2.1. Potentiometric and/or pH Measurements

OCP values were recorded continuously with a data acquisition device (Keithley Instruments, model 2700, Cleveland, OH, USA). Three different reference electrodes were used: (i) An internal reference electrode of a combined pH electrode (Fischer brand, AgCl/Ag/KCl sat./AgCl sat. E = 197.0 mV/SHE); (ii) SCE inserted in a lugging capillary containing KCl 3 M (SCE/KCl sat. E = 244.4 mV/SHE); and (iii) An MSE electrode inserted in a lugging capillary containing saturated K_2_SO_4_ (MSE/K_2_SO_4_ sat. E = 640 mV/SHE). This lugging capillary introduces a junction potential of 1 mV at 25 °C. All potential values were converted with respect to the standard hydrogen electrode (SHE). During experiments, pH was also monitored with a commercial glass electrode that was calibrated daily using commercial standard buffer solutions (4, 7 and 10).

#### 3.2.2. Conductivity Measurements

GEIS measurements used the previously defined PAR 273A. Measuring of the conductivity with the multi-parameter probe was based on the same principle as a 4-pole conductivity meter, which applies a known alternating current (AC) value of 10 µA between two electrodes in the frequency range from 0.1 to 10^5^ Hz and measures the induced potential between two other electrodes. From these two parameters, the resistance of the solution (R) is obtained via the ohm law. The solution resistivity (ρ), which is the reciprocal of the conductivity, σ = 1/ρ, was determined from the relation R_(Ω)_ = ρ_(Ω×m)_ × l_(m)_/S_(m²)_, where l_(m)_/S_(m²)_ represents the geometric factor *k* (m). Since this factor *k* depends on the inter-electrode distance, it has to be determined for each electrode couple studied.

**Figure 3:**
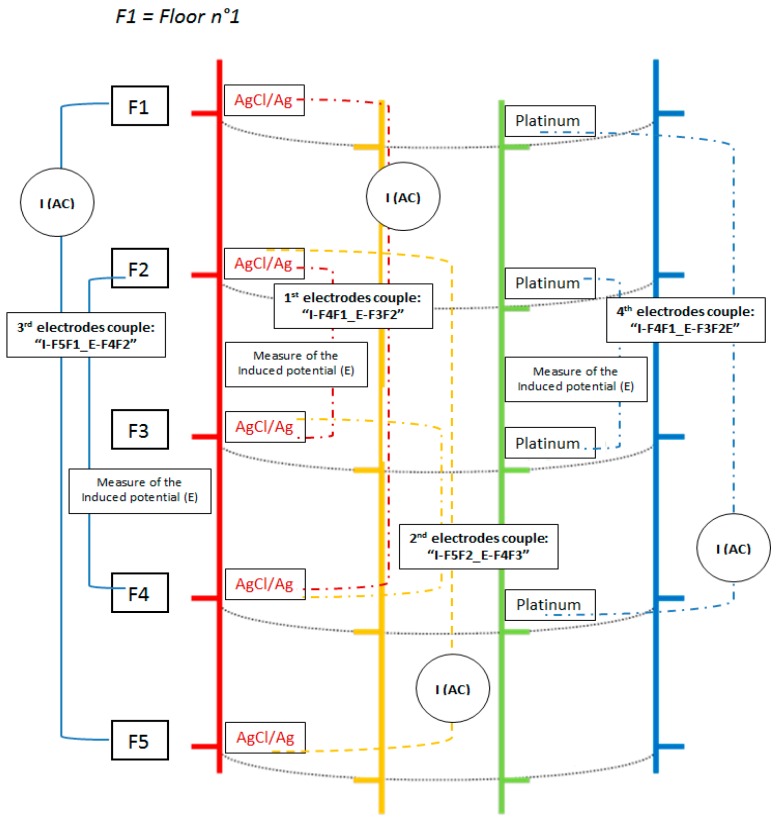
Schematic representation of the multi-parameter probe as well as the different electrode couples used for conductivity measurements.

A schematic representation of the multi-parameter probe with the electrodes used for measuring the conductivity is shown on Figure 3. This also shows the four electrode couples that were tested measuring the conductivity. The first electrode couple is I-F4F1_E-F3F2 (AgCl/Ag-based electrodes); the second is I-F5F2_E-F4F3 (AgCl/Ag-based electrodes); the third is I-F5F1_E-F4F2 (AgCl/Ag-based electrodes); and the fourth is I-F4F1_E-F3F2 (Pt electrodes). The letter “I” is associated to the electrodes used for current injection and the letter “E” is associated to those used for measuring the induced potential. 

#### 3.2.3. Geochemical Modelling

The PHREEQC^®^ (USGS, Denver, CO, USA) geochemical code (see also the PHREEQC web site: https://wwwbrr.cr.usgs.gov/projects/GWC_coupled/phreeqc/) was used for thermodynamic investigation of the E_h_ of the measured sample with the appropriate associated THERMODEM^®^ [58] thermodynamic database generated by BRGM (Orléans, France). Redox potential values were calculated by using speciation data provided by UV-spectrometry (ISO 6332:1988) [59].

## 4. Results

### 4.1. All-Solid-State Monocrystalline Antimony pH Electrode 

The performances of the monocrystalline antimony electrode (Sb(s)) were investigated by monitoring OCPs on pH values ranging from 5 to 12, in accordance with those anticipated in the COx formation once radioactive waste is buried. The reliability and robustness were investigated in the presence of several anions such as chloride, phosphate, nitrate and hydrogenocarbonate/carbonate, since these are or are susceptible to be present on site and are likely to cause OCP drift. Throughout the 18 months of experiments, monocrystalline antimony electrodes were used without any treatment. For a better insight, data were compared to those acquired with antimony-based screen-printed electrodes (Sb(SPEs)) that were renewed every 48 h. First, several experiments were conducted in ammonia pH buffer solutions from pH 7.3 to 11, which includes the major pH values that are anticipated in the COx formation during its evolution as radioactive waste repository. The influence of either chloride (NaCl, 0.1 M) or nitrate (NaNO_3_, from 1.0 × 10^−3^ to 6.0 × 10^−3^ M) was investigated in the same way. 

Afterwards, phosphate pH buffers were used to calibrate the electrodes from pH 5.5 to 7.7. To investigate their influence, measurements made in a phosphate pH buffer solution were compared to those made in an ammonia pH buffer solution. Because the pH of the clay-rock’s pore-water is controlled by carbonate-system equilibria, electrodes were also tested in hydrogenocarbonate/carbonate buffers of various ionic strengths (IS from 0.05 to 0.2 M), from pH 9.2 to 10.2.

The influence of oxygen was investigated by comparing experiments run at atmospheric pressure to experiments run in the glove box, comparing the effect of oxygen on antimony and platinum electrodes. Finally, the monocrystalline antimony electrode was immersed in the reconstituted COx pore-water solution during one month in the glove box. The average potential recorded during this month was compared to our calibration curves, to investigate the reliability and the accuracy of the electrode.

### 4.2. Influence of Anions at Atmospheric Pressure

#### 4.2.1. Feasibility Study and Influence of Chloride on the OCP of Antimony-Based Electrodes 

**Figure 4:**
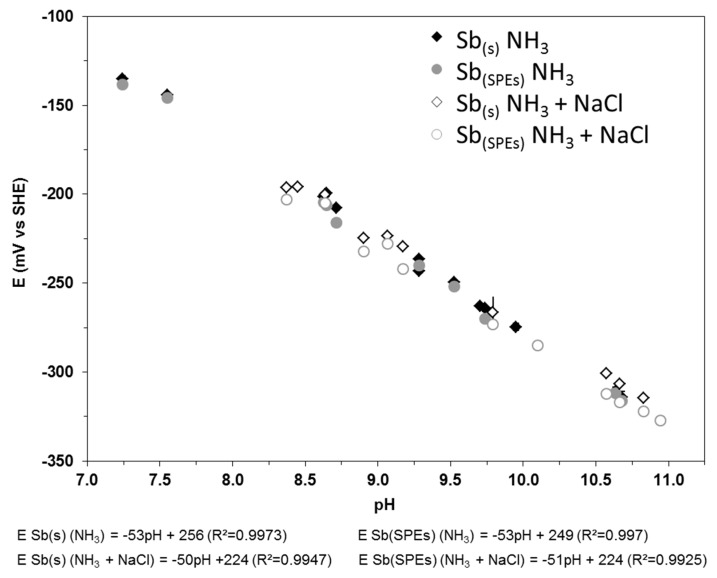
Eh-pH diagram of both the monocrystalline antimony electrode (Sb(s)) and antimony-based screen-printed electrodes (Sb(SPEs)) in ammonia pH buffer solutions, in the absence or presence of 0.1 M NaCl (25 °C, at atmospheric pressure).

The influence of chloride on the antimony electrode OCP was investigated in ammonia pH buffer solutions. Results obtained from both the monocrystalline antimony electrode and antimony-based screen-printed electrodes are shown on Figure 4.

The general convergence of the stabilization potential of the two types of electrode showed that an equilibrium state was reached under the experimental conditions. The potential-pH dependence of all electrodes over the investigated range is similar and linear. The fact that there are no significant differences between the monocrystalline-antimony and screen-printed electrodes demonstrates the reliability and robustness of the monocrystalline-antimony electrode. Overall, results agree with previous studies on the potential-pH relationship of antimony electrodes [8,20,33]: Ives [33] obtained E° = 0.245 V and Glab et al. [8] obtained a slope of 52 mV/pH. The Eh-pH slopes obtained from Sb(s) and Sb(SPEs) are both close to the theory (−59.1 mV/pH, according to Nernst equation, T = 25 °C [9]). As Sb spontaneously oxidizes in air or in water in the presence of oxygen, the electrode potential should be governed by the Sb_2_O_3_/Sb couple (E°(Sb_2_O_3_/Sb) = 0.152 mV/SHE). Our results confirm the metal-cell action theory of metal corrosion [8,33,55] that mentions that a small portion of the antimony electrode surface is the siege of oxygen reduction, causing positive drift of the antimony electrode potential (E°(O_2_/H_2_O) = 1.23 V/SHE). Those results not only show that monocrystalline Sb is an electrode material of interest for measuring pH, but also that it is not affected by the presence of chloride in solution, which agrees with previous results obtained by Uhl and Kestranek in 1923 [18].

#### 4.2.2. Influence of Nitrate on the OCP of Antimony-Based Electrodes 

**Figure 5:**
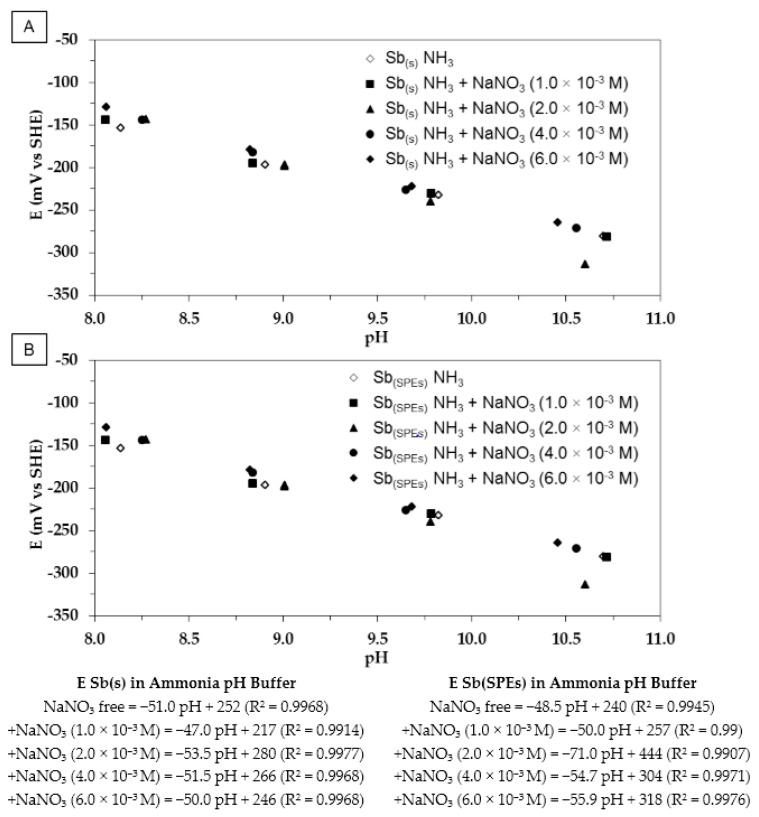
Eh-pH diagram of both Sb(s) (**A**) and Sb(SPEs) (**B**). Experiments were done at 25 °C, at atmospheric pressure, in ammonia pH buffer solutions (IS = 0.1) containing NaNO_3_ (0, 1.0 × 10^−3^, 2.0 × 10^−3^, 4.0 × 10^−3^ and 6.0 × 10^−3^ M).

The presence of oxidizing reagents in solution is highly likely to induce drift in electrode potential. Consequently, experiments tested both Sb(s) and Sb(SPEs) electrodes in the presence of different amounts of nitrate (NO_3_^−^, N^(+V)^) in solution. Nitrate was chosen as oxidizing reagent since it is present in bituminized sludge as nitrate salts, such as NaNO_3_. Bituminized sludges are going to be stored as long-lived intermediate-level radioactive waste within the COx formation and could diffuse in the long-term. The results are shown on Figure 5A,B.

Antimony electrodes (Sb(s) and Sb(SPEs)) were not significantly influenced by the presence of nitrate. The monocrystalline antimony electrode behaves in the same way as in ammonia (with or without chloride) pH buffer solutions, i.e., its Eh-pH slopes as well as its standard potential remain in the same range of values. Conversely, Eh-pH slopes and standard potential values measured from antimony-based screen-printed electrodes show a tendency to increase in the presence of nitrate. The higher OCP value of the Sb(SPEs) over the whole pH range can be explained by interfacial Sb oxidation via NO_3_^−^ such as:

(1) $${\mathrm{NO}}_{3}^{-}+2H^{+}+2e^{-}↔{\mathrm{NO}}_{2}^{-}+H_{2}O$$

(2) $${E^{\prime }{}^{\circ}}_{{\mathrm{NO}}_{3}^{-}/{\mathrm{NO}}_{2}^{-}}=0.4+\frac{\mathrm{RT}}{F}\times \ln\left(10^{-\mathrm{pH}}\right)$$

(3) $${\mathrm{Sb}}_{2}O_{3\left(s\right)}+6H^{+}+6e^{-}↔2{\mathrm{Sb}}_{\left(s\right)}+3H_{2}O$$

(4) $$3{\;\mathrm{NO}}_{3}^{-}+2{\mathrm{Sb}}_{\left(s\right)}↔{\mathrm{Sb}}_{2}O_{3\left(s\right)}+3{\mathrm{NO}}_{2}^{-}$$

A small Sb(SPEs) part of the antimony electrode surface is the seat of nitrate reduction, which leads to a slight positive drift of the OCP. The measured values can be interpreted as a mixed potential between Sb_2_O_3(s)_/Sb_(s)_ and NO_3_^−^/NO_2_^−^ redox couples. These results demonstrate the robustness and the usefulness of the massive monocrystalline antimony electrode for monitoring pH within repositories dedicated to nuclear waste disposal.

#### 4.2.3. Influence of Phosphate on the OCP of Antimony-Based Electrodes

**Figure 6:**
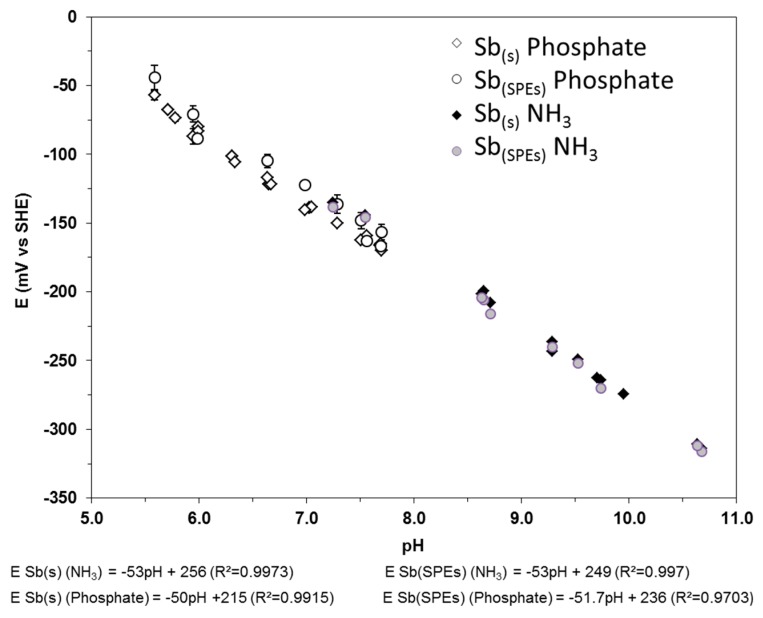
Eh-pH diagram of both the Sb(s) and Sb(SPEs) acquired at 25 °C, at atmospheric pressure, in phosphate and ammonia pH buffer solutions (IS = 0.1).

The influence of the presence of phosphate P^(V)^ on the electrode OCP was studied by means of phosphate buffer species (NaH_2_PO_4_/Na_2_HPO_4_ − IS = 0.1) from pH 5.6 to 7.7, comparing the data to those obtained from ammonia chloride free pH buffer solutions. The results are shown on Figure 6.

The electrodes present a near-Nernstian behaviour over the investigated range. The standard potential is slightly lowered in the presence of phosphate. One of the early investigations of phosphate influence was made by Gysinck [60], who observed a kink in the calibration curve between pH 7.1 and 8.2. Green and Giebish [29] and Perley [28] also investigated the influence of phosphate on the Sb electrode, and observed electrode potential drift. Nevertheless, our results show that the presence of phosphate species does not significantly influence the analytical response of the Sb electrodes. Work carried out by Glab et al. (1981) [16] is in better agreement with our results, showing very little dependence of the Sb electrode to phosphate concentration.

#### 4.2.4. Influence of Carbonate and Ionic Strength on the OCP of Antimony-Based Electrodes 

**Figure 7:**
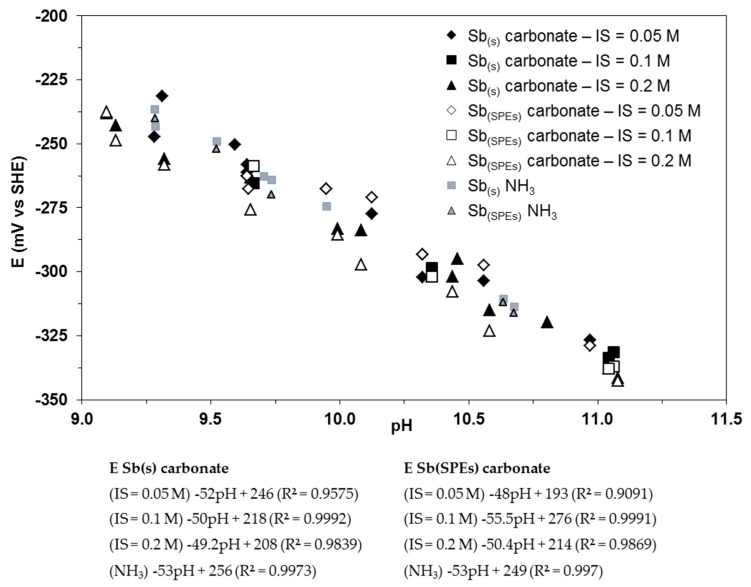
Eh-pH diagram of both Sb(s) and Sb(SPEs). Experiments were run in carbonate pH buffer solutions with various ionic strength (IS) and ammonia pH buffer solution under atmospheric pressure at 25 °C.

In natural water samples, ionic strength is a variable chemical parameter [61]. Its influence on Sb-based-developed electrodes was investigated in NaHCO_3_/Na_2_CO_3_ at different ionic strengths ranging from 0.05 to 0.2 M. The results are compared to those obtained in ammonia pH buffer solutions and shown on Figure 7. Measurements in a carbonate pH buffer solution at different ionic strengths are close to those measured in ammonia buffers, demonstrating the insignificant influence of carbonate species on electrode OCP. Green and Giebisch [29] studied the influence of ionic strength on a micro antimony electrode by means of a phosphate buffer and showed that, independently of hydrogen activity, electrode potential variations occurred when varying the ionic strength. Our results are in better agreement with those published by Caflisch et al. [12], demonstrating that antimony electrodes are not quantitatively influenced by the presence of hydrogenocarbonate/carbonate anions in solution (from 0.05 to 0.2 M).

All our results show that the potential of a massive monocrystalline antimony electrode is not influenced by chlorides (from 0 to 0.1 M), or phosphates (from 0 to 0.1 M), or nitrates (from 0 to 6 mM), or carbonates (from 0 to 0.2 M), demonstrating its robustness and thus its interest for pH monitoring within repositories dedicated to nuclear radioactive waste disposal, such as the one at Bure in France. Moreover, no hysteresis effects affected the antimony electrodes. 

Overall, our results demonstrate the interest of screen-printed electrodes for generic studies of electrode materials. It also should be noted that the antimony-based screen-printed electrodes made for this study present a better behaviour than other Sb-based screen-printed electrodes described in the literature [8,32]. Koncki and Mascini [32] mentioned a certain irreproducibility of antimony-based sensors made by screen-printing as well as hysteresis effects. Our antimony-based SPEs were renewed every 48 h and, as shown by the different calibration curves, there are no doubts regarding the reproducibility of the measurements or the absence of hysteresis effects. Moreover, our results in various buffers showed no effects of complexing ligands as was the case for some authors [8,16,32]. Compared to the −40/−45 mV/pH sensitivity obtained by Koncki and Mascini [32] under specific conditions (i.e., the only suitable results were obtained in TRIS buffer and in the absence of chloride), the sensitivity of our antimony-based SPEs is around −50/−55 mV/pH and does not depend on the nature of buffer species. Our antimony-based SPEs show a near-Nernstian response (an average of −53.6 mV/pH) and thus appear to be suitable electrodes for accurate pH measurements. As pH sensors, the performance level of Sb-based screen-printed electrodes can be compared to that obtained with CeO_2_-based [38] or RuO_2_-based [32] SPEs.

### 4.3. Calibration Curve of the Monocrystalline Sb Electrode at Amospheric Pressure and in the Glove Box. Comparison with a Pt Electrode. Investigation of Measurement Accuracy in the Reconstituted COx Pore Water

**Figure 8:**
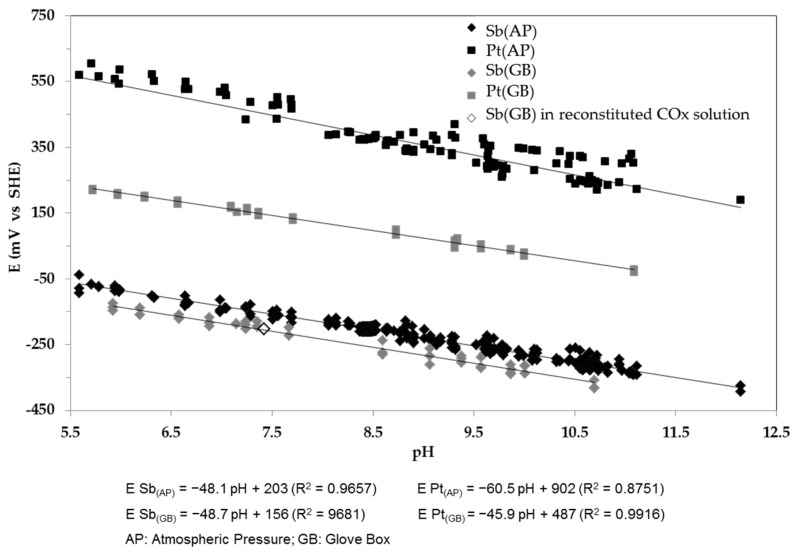
Comparison of calibration curves obtained from antimony (Sb(s) and Sb(SPEs)) and platinum electrodes obtained at 25 °C, under atmospheric pressure and in the glove box. An antimony potential value (vs. SHE) representing the average potential of the electrode in the reconstituted COx solution over one month was added.

In the same way as the experiments conducted at atmospheric pressure, experiments were also performed in the glove box. In parallel and for comparison, the same measurements were performed with four Pt electrodes. The results are presented on Figure 8, where “Sb” represents all data obtained from Sb(s) and Sb-based SPEs.

As well as Sb electrodes, Pt electrodes present a Nernstian behaviour as a function of pH at atmospheric pressure. In absence of any other redox couple except O_2_/H_2_O, the potential of the Pt electrode is governed by the PtO/Pt couple (E° (PtO/Pt) = 900 mV/SHE).

Measurements acquired with Pt electrodes are in good agreement with published data [43,61]. At a given pH at atmospheric pressure and in the absence of any species able to fix the redox potential except O_2_/H_2_O (PO_2_ ≈ 0.2 atm), a difference of 700 mV (at pH 0) exists between E°_PtO/Pt_ and E°_Sb__2__O__3/__Sb_. This difference is reduced to about 330 mV in the glove box. The Sb-based sensitivity to pH change is not affected (−48.1 pH vs. −48.7 pH) with regard to the investigated conditions. The intercept at pH = 0 differs by approximately 50 mV, demonstrating the low dependence of the Sb electrode versus oxygen and, consequently, the interest of electrodes based on Sb for pH measurement.

To verify the reliability of a Sb(s)-based electrode in our context of pH monitoring within a radioactive waste-disposal site, the monocrystalline antimony electrode was tested for about one month in the reconstituted COx pore-water solution in the glove box. Measurements using the four Pt electrodes were done every 5 min in the same reconstituted COx solution, for a total of 8640 measures over the month. Average pH values determined from the Sb(s) electrode and Pt electrodes are summarized in Table 3.

The average potential of the Sb-electrode over one month was added on Figure 8 (−203.1 mV vs. SHE). The calculated standard deviation is low (2.9 mV), again demonstrating the robustness of the electrode. From the figure it is clear that the measured potential over one month in the reconstituted COx solution in the glove box agrees very well with our calibration curve and the same is true for the Pt electrodes whose average potential is also very stable (151.2 ± 2.88 mV/SHE).

Concerning the relative standard deviation of the commercial pH electrode, the monocrystalline Sb electrode and the platinum electrodes, the measured pH values are in line with our expectations, which confirms the reliability and so the interest of the monocrystalline Sb electrode for monitoring pH within a future radioactive waste disposal site.

### 4.4. Investigation of Inert Pt, Au and GC Electrodes for Redox Potential Measurements

**Figure 9:**
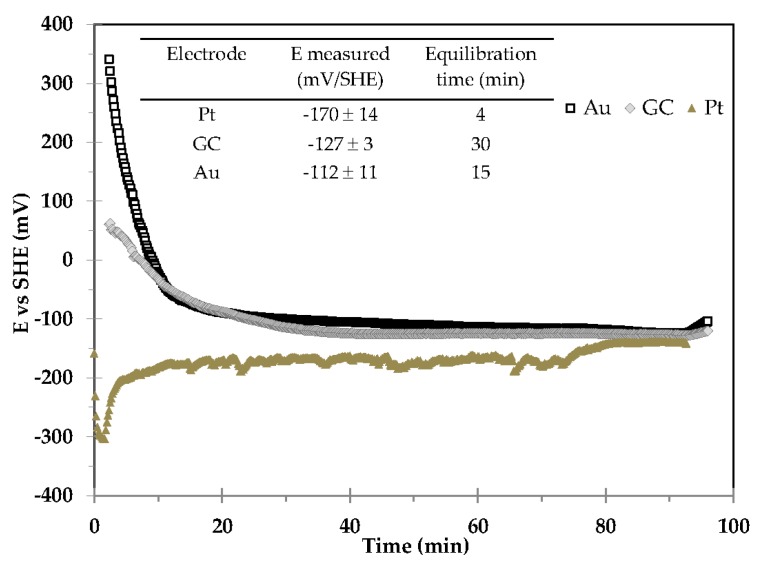
Evolution of Au, GC and Pt OCP electrodes as a function of time. The experiment was conducted in 0.1 M NaCl solution containing 100 mg of Fe° powder at pH 8 and 25 °C, in the glove box.

This experiment consisted in investigating the behaviour of gold, glassy carbon and platinum electrodes in samples where iron dominated redox reactions. The investigation was conducted in a 0.1 M NaCl solution containing 100 mg of Fe° powder at pH 8 (not balanced system, Figure 9) in the glove box. Prior to measurements, the solution was stirred during 24 h.

#### 4.4.1. Electrode Performances and Robustness—Inert Electrodes

Potentials recorded by all immersed inert electrodes converge to a value of the same order of magnitude. This fact demonstrates that, under the same experimental conditions, the same equilibrium state has been reached on all inert electrodes. 

#### 4.4.2. Comparison between Measured Potential, Speciation Measurements and Geochemical Modelling

At pH 8, the redox couple fixing the potential should be H^+^/H_2(g)_. There is no thermodynamical equilibrium between Fe° and H_2_O, as Fe° corrodes under anaerobic conditions. In the presence of O_2_, Fe° also corrodes to form Fe^2+^, which can then be oxidized in Fe^(3+)^ that can precipitate. Thus, Fe^(III)^/Fe^(2+)^ progressively becomes the predominant redox couple leading to a progressive increase of the redox potential. The presence of dissolved Fe^(3+)^ (provided by speciation measurements obtained by UV-visible spectroscopy (Fe^(3+)^ 3 × 10^−7^ M, Fe^(2+)^ 2.95 × 10^−6^ M) corroborates the presence of oxygen. The convergence to a stabilized potential close to −140 mV/SHE at pH 8 and 25 °C (Figure 9) demonstrates the limitation of the O_2_ intrusion (absence of total oxidation), and the qualitative agreement between the acquired OCPs of all inert electrodes argues for an influence of O_2_ trace concentrations originating from the gas phase in the glove-box. Considering the disturbance by O_2_ in the sample, the geochemical modelling predicts:

1. E_h_ = −165 mV/SHE in the presence of lepidocrocite + 0.10 × 10^−6^ M Fe^2+^ in solution.
2. E_h_ = −157 mV/SHE in the presence of goethite + 0.07 × 10^−6^ M Fe^2+^ in solution.

Nevertheless, problems of solubility uncertainty are assumed in the database. The redox potential was probably fixed by the Fe(OH)_3_/Fe^2+^ redox couple, which depends upon Fe(OH)_3_ solubility as a function of pH. While limitations to the interpretation of E_h_ remain, the interest in continuous monitoring of voltage measurements using multiple redox electrodes is clearly shown in order to ensure reliable qualitative measurements.

This example highlights that for not well-balanced oxygen-sensitive systems, speciation should absolutely be preserved to provide qualitative information on redox conditions. Nevertheless, preserving the initial conditions is extremely difficult. A complementary approach by successively coupling amperometric and potentiometric measurements, in which an electro-active redox mediator improved the rate of electron transfer from the redox active solid phases to the electrode, has been developed for redox-potential measurements of minerals [62].

### 4.5. All-Solid-State AgCl/Ag- and Ag_2_S/Ag-Based Electrodes as Reference or Selective Electrodes

The long-term behaviour of AgCl/Ag- and Ag_2_S/Ag-based electrodes was investigated in the reconstituted COx pore water in the glove box, at constant pH (7.4) and by varying the pH by means of addition of NaOH and K_2_CO_3_ solutions in the range 7.4 to 11. Measurements were made with respect to MSE.

#### 4.5.1. Experiments Performed at Constant pH (7.4)

**Figure 10:**
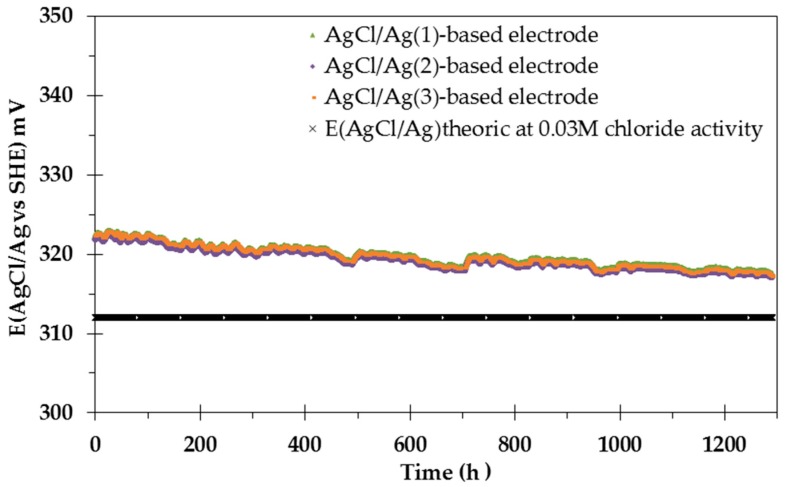
Evolution of the potentiometric response of the all-solid-state AgCl/Ag-based electrodes at constant pH (7.4) as a function of time over 54 days. Measurements were made in the reconstituted COx solution, at 25 °C in the glove box.

Figure 10 shows the potentiometric response of three AgCl/Ag/[Cl^−^] = 0.04 M electrodes over almost two months in the reconstituted COx solution at a constant pH of 7.4. It also shows the theoretical OCP calculated from the Nernst equation by considering a chloride concentration of 0.04 M as well as the ionic strength of the solution (I = 0.1 M). The activity coefficient of chloride (γ_Cl-_) was calculated from the Debye-Hückel model and is equal to 0.75. An increase of chloride-ion activity leads to a decrease of the AgCl/Ag-based electrode potential, according to the Nernst equation.

The electrochemical behaviour at zero current of the three AgCl/Ag/[Cl^−^] = 0.04 M electrodes is very similar. A very slight decrease of the potential is observed over time: 4 mV loss over 1200 h for all electrodes, corresponding to an increase in chloride activity of 14.4%. This small deviation is consistent with the 13.7% water loss measured in the cell due to evaporation, leading to an increase of the chloride activity over time.

In comparison to the behaviour observed at constant pH when using a solid-state AgCl/Ag/KCl reference electrode based on carbon nanotubes and polyacrylate membranes [63], our results obtained on massive AgCl/Ag/[Cl^−^] = 0.04 M electrodes thus showed encouraging results: 4 mV loss over 1200 h due to evaporation compared to an average 1 mV loss per hour obtained by [63]. The potential response of AgCl/Ag/[Cl^−^] = 0.04 M electrodes when subjected to pH variations was then investigated.

#### 4.5.2. Experiments Performed under pH Variations

**Figure 11:**
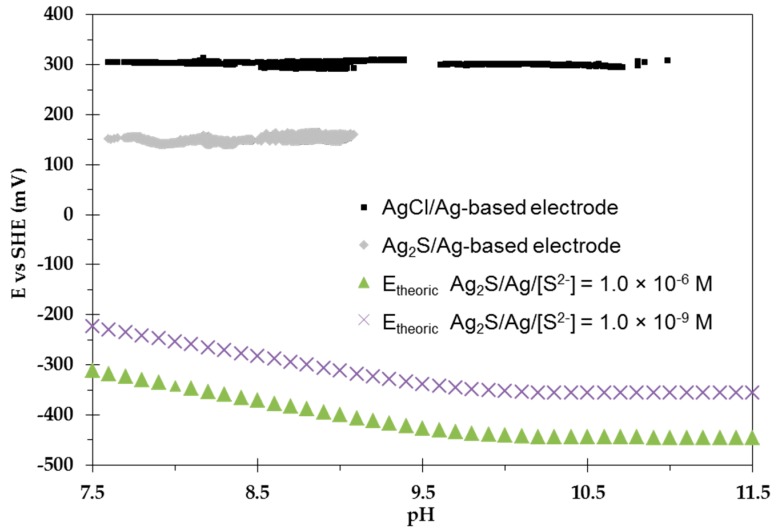
Eh-pH diagram of both AgCl/Ag- and Ag_2_S/Ag-based electrodes. Experiments were done in the reconstituted COx solution at 25 °C, in the glove box. NaOH was used to increase pH. Theoretical Eh-pH variation curves of the Ag_2_S/Ag-based electrode in the presence of [S^2−^] at total concentrations of 1.0 × 10^−6^ M and 1.0 × 10^−9^ M were added for comparison.

To investigate the influence of pH on the potentiometric response of AgCl/Ag/[Cl^−^] = 0.04 M electrodes, NaOH was added continuously to the reconstituted COx solution. At the same time, another experiment was conducted on an Ag_2_S/Ag-based electrode (Figure 11).

As well as at constant pH, the potential of the AgCl/Ag/[Cl^−^] = 0.04 M electrodes was not significantly influenced by pH variations. This is in good agreement with the theoretical electrode behaviour (19) and encourages their utilization as reference electrodes for further experiments such as to calibrate the electrodes for monitoring the near field of a nuclear waste disposal site.

In comparison to AgCl/Ag/[Cl^−^] = 0.04 M, the Ag_2_S/Ag-based electrode is characterized by an OCP that is constant (E = 151 ± 4.6 mV) in the investigated pH range. 

Similar to the AgCl/Ag/[Cl^−^] = 0.04 M electrodes, the results obtained from an Ag_2_S/Ag-based electrode are encouraging for further experiments in the presence of S^(−II)^.

### 4.6. Measurements of the Conductivity in Solution from the Multi-Parameter Probe 

Multiplying electrode couples for accurate conductivity measurements appears to be crucial. For this purpose, the accuracy of the conductivity measurements was investigated on the four electrode couples I-F4F1_E-F3F2(AgCl), I-F5F2_E-F4F3(AgCl), I-F5F1_E-F4F2(AgCl) and I-F4F1_E-F3F2(Pt), as shown on Figure 3.

Prior to any measurement on a real sample, it is necessary to determine the geometric factor (k) of each investigated electrode couple. The calibration step of the multi-parameter probe was done by measuring the impedance (|Z|) of four sodium chloride solutions whose concentrations ranged from 10^−4^ to 10^−1^ M. The resistivity values of these solutions were measured by means of a commercial conductivity probe, and are reported in Table 4.

The influence of the frequency of the AC current applied between the two injection electrodes was investigated by calibrating the four electrode couples at various frequencies ranging from 0.1 to 10^5^ Hz. The objective of this investigation was to determine the optimal frequency range for measuring the conductivity in the range from 10^−4^ to 10^−1^ M (i.e., from 1.28 × 10^−3^ S·m^−1^ to 1.22 S·m^−1^).

#### 4.6.1. Influence of the Alternating Current Frequency

**Figure 12:**
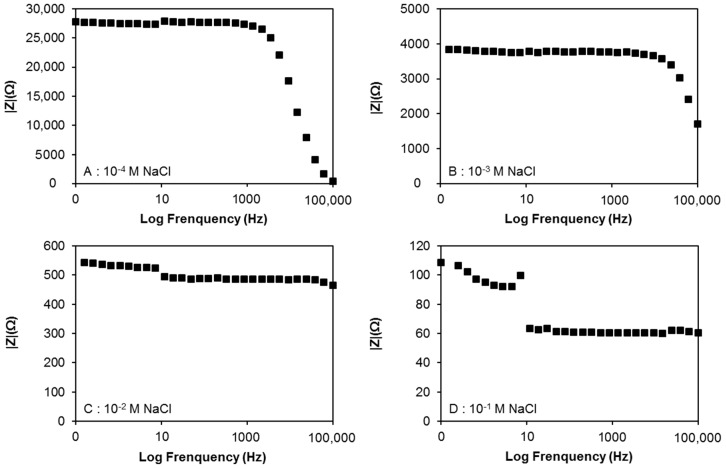
Variation of the impedance values as a function of alternating current frequency resulting from measurements carried out on the second electrode couple (I-F5F2_E-F4F3_AgCl) in sodium chloride solutions of different concentrations: (**A**) 10^−4^ M, (**B**) 10^−3^ M, (**C**) 10^−2^ M, (**D**) 10^−1^ M.

In order to investigate the frequency influence on our experiments, impedance measurements were carried out at different frequencies ranging from 0.1 to 10^5^ Hz for each of our electrode couples and in each sodium-chloride solution (10^−4^, 10^−3^, 10^−2^, 10^−1^ M). The results and conclusions being the same for all electrode couples, only the one obtained from the second electrode couple (I-F5F2_E-F4F3_AgCl) will be discussed (Figure 12).

As shown on Figure 12, impedance values measured in low-conductive solutions (Figure 12A,B) are fairly stable at low frequencies, but vary significantly at high frequencies. The inverse is observed in more conductive solutions (Figure 12C,D), where the impedance values are fairly stable at high frequencies, but not at low frequencies. This shows that the less the solution is conductive, the lower the frequency should be. As it was decided to calibrate the device in a concentration range of 10^−4^ M to 10^−1^ M, i.e., to determine a geometric factor “*k*” that would allow the conversion of impedance (|Z|) into resistivity (ρ) over this concentration range, the alternating current (AC) frequency had to be chosen carefully, considering the impedance variation as a function of AC frequency. For instance, regardless of the concentration of the solution, the impedance remains constant as a function of the frequency from 10 Hz to about 1500 Hz (Figure 12). Therefore, the geometric factor was determined with a 1373 Hz AC frequency (10 µA). The determination and comparison of the geometric factors of each electrode couple is presented in the next section.

#### 4.6.2. Determination of the Geometric Factors 

By applying the procedure described earlier in the Section 4.6 (Measurements of the conductivity in solution from the multi-parameter probe), the geometric factors of each electrode couple were calculated. Table 5 summarizes the measurements at different concentrations ranging from 10^−4^ to 10^−1^ M. The geometric factor "k" was obtained by plotting the resistivity of the solutions (Table 5) as a function of impedance, since it corresponds to the slope. 

Comparing the geometric factors obtained from couples 1 and 2 (AgCl/Ag-based electrodes) shows that measurement reproducibility is good. 

Comparing the results obtained with silver chloride/silver electrodes and platinum electrodes, i.e., couples 1, 2 and 4, shows that the geometric factor does not depend on the type of electrode. 

Finally, comparing the results of electrode couple 3 with those of the other electrode couples shows that the geometric factor value depends on the distance between electrodes. However, this dependence on the distance between electrodes seems only to concern the distance between electrodes for measuring the induced potential. As shown on Table 5, when the distance between these electrodes is doubled, the geometric factor increases by a factor of two as well. 

Geometric factors were also calculated for a lower AC frequency (79 Hz) following the same approach as above. The results (not presented) are very similar to those just described, showing a relative gap of the geometric factors ranging from 0.75 to 3%, depending on the electrode couple considered. The calibration of the device allowed measuring the resistivity of the reconstituted COx pore water from impedance measurements at 1373 Hz. The good agreement between the conductivity value measured with the multi-parametric probe (0.78 ± 0.02) S·m^−1^, the mean value of the data acquired by the four electrode couples) and that acquired with a conventional four-cell conductivity electrode (0.85 S·m^−1^) shows the accuracy of our new probe (relative gap of 8.2%), which can be used for punctual conductivity measurements. It can also be used for establishing a vertical conductivity profile of the solution flowing through the multi-parametric probe, by injecting the current between floors 1 and 5, and by successively measuring the induced potential between floors 5–4, 4–3, 3–2 and 2–1. If the conductivity of the solution changes, then the flow of the solution through the device can be estimated.

## 5. Conclusions

We present an innovative electrochemical device for pH, redox potential and conductivity monitoring in near-field rock of deep geological radioactive waste repositories. A monocrystalline antimony electrode was tested over 16 months. Both reliability and robustness of this electrode were clearly demonstrated for pH monitoring in the range of 5.5–12 within a radioactive nuclear-waste disposal site. 

OCP measurements provided by Pt, Au and glassy carbon (GC) electrodes for determining the E_h_ value confirmed the robustness of platinum as an indicator electrode for this purpose. Voltage measurements by gold and GC electrodes tended towards those provided by platinum ones (same order of magnitude), demonstrating the analytical feasibility of redox measurement via other inert electrodes. 

All-solid-state AgCl/Ag/[Cl^−^] = 0.04 M electrodes showed a constant OCP value (E = 319.2 ± 1.4 mV) over one month of analyses of reconstituted COx pore water in the 7.4–11 pH range. Under the investigated conditions, the electrodes did not show potential drift when subjected to pH variations which opens the possibility of using them as reference electrodes. Under the same conditions and in the absence of S^(−II)^, the OCP of an Ag_2_S/Ag-based electrode acquires the constant OCP value of E = 151 ± 4.6 mV in the pH range of 7.4 to 9. 

The multi-parametric device can also be used for conductivity measurements by GEIS. The good agreement between the conductivity value measured with the multi-parametric probe (0.78 ± 0.02 S·m^−1^), which is a mean of the data acquired by the four electrode couples, and the one acquired with a conventional four-cell conductivity meter (0.85 S·m^−1^) testifies to the accuracy of the method. 

Overall, the bundle of electrodes as designed by us appears suitable for monitoring the COx formation during its envisaged use for hosting a nuclear waste repository. Further is ongoing to develop abacuses for an accurate calibration of the new probe. Experiments for estimating corrosion rates of the different electrode materials in the reconstituted COx solution are planned as well.

## Figures and Tables

**Table 1:** The different buffer species with their effective pH range.

Buffer Species	Effective pH Range
NH_4_^+^/NH_3_	7.2–11.0
HCO_3_^−^/CO_3_^2−^	9.1–11.1
H_2_PO_4_^−^/HPO_4_^2−^	5.5–7.8

**Table 2:** Composition of the reconstituted COx solution at 25 °C.

Species in Solution	Concentration (M)	Species in Solution	Concentration (M)
Ca^2+^	0.0074	SO_4_^2−^	0.0156
Mg^2+^	0.0067	Cl^−^	0.0400
Sr^2+^	0.0002	Total Carbon	0.0032
Na^+^	0.0450	pH	7.0–7.4
K^+^	0.0010	Ionic strength	0.1

**Table 3:** Average pH and OCP of Sb electrode measured over 1 month in the reconstituted COx solution in the glove box.

Electrode	Average Value (pH Units)	Standard Deviation
Commercial pH electrode	7.42	±0.03
Monocrystalline Sb-electrode	7.37	±0.06
Pt electrodes (mV/SHE)	7.32	±0.07

**Table 4:** Conductivity and resistivity values of the four sodium-chloride solutions used to determine the geometric factor value of each electrode couple.

NaCl (M)	σ (S·m^−1^)	ρ (Ω·m)
10^−1^	1.2155	0.8227
10^−2^	0.12	8.3333
10^−3^	0.0126	79.3651
10^−4^	0.001274	784.9294

**Table 5:** Summary of the impedance measures realized on the different electrodes couples for the determination of the geometric factors. An AC of 10 µA at a frequency of 1373 Hz has been applied during these experiments.

N° Couple	Injection Electrodes (I)	Induced Potential Electrodes (E)	|Z| (Ω) at Different NaCl Content (M) and at Frequency = 1373 Hz				*k* (m^−1^)
			10^−1^ M	10^−2^ M	10^−3^ M	10^−4^ M	
1	F4F1_AgCl	F3F2_AgCl	58.98	475.55	3684.48	26,849.22	0.0296
2	F5F2_AgCl	F4F3_AgCl	60.37	484.18	3736.20	26,975.55	0.0294
3	F5F1_AgCl	F4F2_AgCl	119.39	957.7	7377.56	53,710.79	0.0148
4	F4F1_Pt	F3F2_Pt	58.45	472.20	3661.99	26,417.52	0.0301
